# Histological and mechanical comparisons of arytenoid cartilage between 4 brachycephalic and 8 non-brachycephalic dogs: A pilot study

**DOI:** 10.1371/journal.pone.0239223

**Published:** 2020-09-17

**Authors:** Satoshi Tokunaga, E. J. Ehrhart, Eric Monnet

**Affiliations:** 1 Clinical Sciences, College of Veterinary Medicine and Biomedical Sciences, Colorado State University, Fort Collins, Colorado, United States of America; 2 Ethos Diagnostic Science, San Diego, California, United States of America; University of Bari, ITALY

## Abstract

Brachycephalic airway syndrome (BAS) is a well-established cause of respiratory distress in dogs. BAS without surgical correction results in eventual laryngeal collapse. Arytenoid lateralization has been used to treat severe laryngeal collapse with some highly variable results. Chondromalacia and decreased stiffness of the arytenoid cartilage has been postulated a source of failure after arytenoid lateralization but no report of the histological characteristics and mechanical strength of arytenoid cartilage in brachycephalic dogs has been reported. Here we report histological and mechanical features in arytenoid cartilage of brachycephalic dogs. We identified the arytenoid cartilage in brachycephalic dogs presented degenerative histological characteristics and decreased load to failure and stiffness compared to that in non-brachycephalic dogs. Together, these observations suggest that degenerative condition of arytenoid cartilage in brachycephalic dogs could contribute to chondromalacia and mechanical weakness of arytenoid cartilage and result in cause of failure after arytenoid lateralization.

## Introduction

Brachycephalic airway syndrome (BAS) is a well-established cause of respiratory distress in dogs [1–3]. Primary characteristics of BAS include congenital anatomic abnormalities such as skull confirmation anomalies, soft tissue changes (e.g.; stenotic nares, elongated soft palate), hypoplastic trachea, and nasopharyngeal turbinates. Although no study has been reported to establish exact pathophysiology of laryngeal collapse in brachycephalic dogs these anatomic abnormalities increase airway resistance during inspiration and has been widely considered to lead to development of secondary changes that include severe soft tissue edema of palate and larynx, everted saccule and tonsil, and laryngeal collapse [4, 5]. So far no research has investigated changes in rigidity of laryngeal cartilage or correlation between laryngeal cartilage stiffness and timing of surgical correction for primary BAS components such as stenotic nares or elongated soft palate. However BAS without surgical correction has been thought to result in continuous increased airway resistance, increased negative intra-glottis luminal pressure, increased airway velocity, loss of laryngeal cartilage rigidity, medial deviation of laryngeal cartilage, and eventual laryngeal collapse [6–8]. Torrez et al reported that laryngeal collapse was present in 53% of dogs with BAS and is relatively common in dogs presented for surgical correction of BAS [9]. Laryngeal collapse has been reported even in young brachycephalic dog [8]. Prognosis for dogs with laryngeal collapse is variable depending on its severity. Dogs with stage I laryngeal collapse usually show marked improvement following nasal alaplasty, staphylectomy, and saccule resection. On the other hand, prognosis of stage II and III laryngeal collapse appears to be very guarded although some surgical interventions including aryepiglottic fold resection or permanent tracheostomy have been reported with some limited success. Arytenoid lateralization has been used to treat severe laryngeal collapse with some highly variable results [8–11]. It has been postulated that chondromalacia and decreased stiffness of the arytenoid cartilage is a source of failure after arytenoid lateralization [8–12]. Pink et al [8] reported laryngeal collapse in 7 brachycephalic puppies aged between 4.5 and 6 months which might be related to chondromalacia as a cause of the collapse. However, to the author’s knowledge, there are no reports of the histological characteristics and mechanical strength of arytenoid cartilage in brachycephalic dogs.

The objectives of this pilot study are to compare the histological characteristics and mechanical strength of arytenoid cartilage between brachycephalic and non-brachycephalic dogs. We hypothesized that arytenoid cartilage of brachycephalic dogs have histological degenerative changes and decreased mechanical strength compared to that of non-brachycephalic dogs.

## Materials and methods

This study was approved by the "Clinical Review Board" at Colorado State University: VCS #2018–178 “Histological and biomechanical evaluation of arytenoid cartilages of brachycephalic dogs.

### Sample collection

Arytenoid cartilages were obtained from brachycephalic dogs (BC group) and non-brachycephalic dogs (NBC group) euthanized at Colorado State University Veterinary Teaching Hospital for reasons non-related to the study. The arytenoid cartilage specimens were collected with sharp dissection. The surrounding soft tissues were removed without damaging the cartilage. For each dog, the pair of arytenoid cartilages was randomly assigned to histological analysis or biomechanical analysis. The arytenoid cartilages used for histological analysis were fixed in 10% buffered neutral formalin. The arytenoid cartilages used for biomechanical analysis were covered with saline soaked gauze and stored at -70°C until used. Previous study showed that the similar stiffness and hysteresis properties in articular cartilage were observed between unfrozen controls and samples stored at -20°C as well as those stored at -80°C [13].

### Histological assessment

The fixed samples were paraffin-embedded and 6-μm sections were obtained with a microtome instrument. The sections were stained with H&E to evaluate the overall structure of the cartilage and chondrocytes. Safranin O stain was used to detect proteoglycans and toluidine blue stain was used to assess the degree of metachromasia in the cartilage tissues.

An observer blinded to the test group evaluated H&E stained sections, counted both the total number of chondrocytes and degenerative chondrocytes in 5 random areas at magnification of 400X, and reported the median number of total chondrocytes and degenerative chondrocytes. Degenerative chondrocyte was defined as that exhibit a characteristic pattern of morphologic changes including cell shrinkage, condensation and chromatin condensation and nucleosomal fragmentation [14]. The sections stained with safranin O were graded as; 0: normal, 1: slightly reduced, 2: moderately reduced, 3: none (or almost none) and the sections stained with toluidine blue were graded as; 0: normal, 1: slightly reduced, 2: markedly reduced, 3: no metachromatic change according to the histological grading scale described by previous reports [15–17].

### Mechanical evaluation

**Fig 1:**
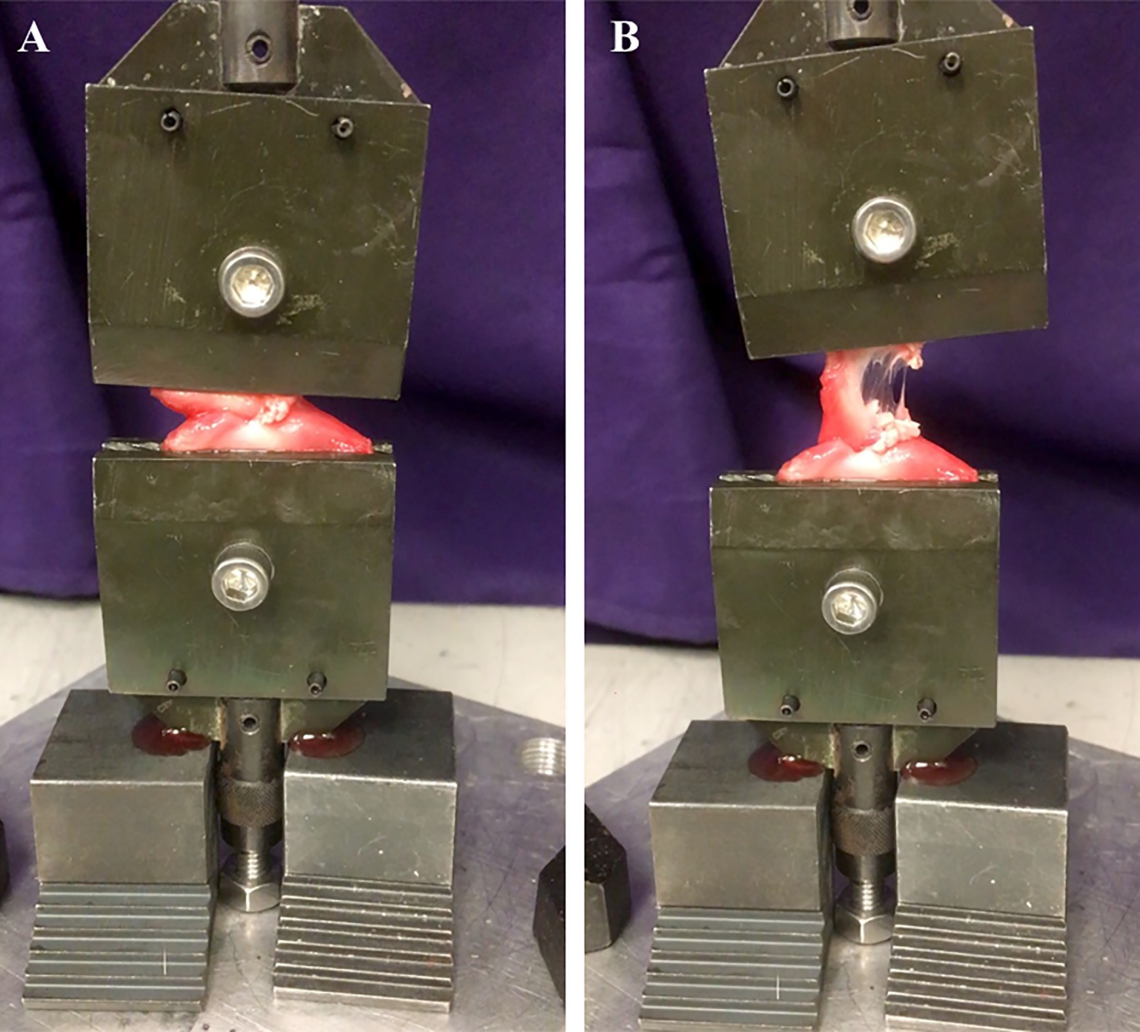
Mechanical evaluation of arytenoid cartilage before (A) and after (B) tensile load was applied.

The load to failure of each arytenoid cartilage sample was analyzed using the universal tensile strength analyzer Mini Bionix (MTS Systems, Eden Prairie, MN). The frozen samples were thawed for 24 hours in the refrigerator at 4°C before mechanical evaluation. The arytenoid cartilage tissue was mounted between the two grips in the apparatus, with muscular process griped by upper grip and cuneiform process gripped by lower grip (Fig 1). The grip surfaces were lined with sand paper to prevent slipping. Each section was stretched at a testing speed of 10 mm per minute. Tensile load was applied continuously until failure. Load to failure was defined as the maximal load or the load peak prior to a decrease in load associated with increasing displacement. Failure loads were measured as units of force in Newtons. The load to failure (N), time to failure (s), and displacement (mm) was recorded and stiffness of the arytenoid cartilage was calculated from the slope of the load-deformation curve in Newtons per millimeter. A linear regression analysis was used to determine the slope.

### Statistical analysis

Histological and mechanical data were compared with a non-parametric test between the BC group and the NBC group, with significance set at p<0.1 because of small sample size (JMP, version 13, SAS Institute Inc, Cary, NC). Data is presented as median and range.

## Results

Arytenoid cartilage samples were obtained from four brachycephalic dogs and eight non-brachycephalic dogs for both histological assessment and biomechanical testing (Table 1). Brachycephalic breeds included Boxer (1), French Bulldog (1), English Bulldog (1), and Pekinese (1). Non-brachycephalic breeds included walker hounds (6), Golden Retriever (1), and German shepherd (1). There were 3 spayed females and 1 castrated male in the BC group. There were 6 intact females and 2 spayed females in NBC group. Median age was 137.5 months (range: 74–180 months) for the BC group and 12 months (range: 12–132 months) for the NBC group (P = 0.0293). Median body weight was 16.75 kg (range: 4.5–24.5 kg) for the BC group and 25.0 kg (range: 25–34.4kg) for the NBC group (P = 0.0037).

**Table 1:** Distribution of brachycephalic dogs and non-brachycephalic dogs.

	Brachycephalic	Non-Brachycephalic	P
Number of samples	4	8	
Breed	Boxer (1) French Bulldog (1) English Bulldog (1) Pekinese (1)	Walker hound (6) Golden Retriever (1) German Shepherd (1)	
Sex	FS (3)	FS (2)	
	MC (1)	FI (6)	
Age (mo)	137.5 (74–180)	12 (12–132)	0.0293
Body weight (kg)	16.75 (range: 4.5–24.5)	25 (range: 25–34.4)	0.0037

Data for age and body weight was presented as median and range.

FI = female, intact. FS = female, spayed. MC = male, castrated.

**Fig 2:**
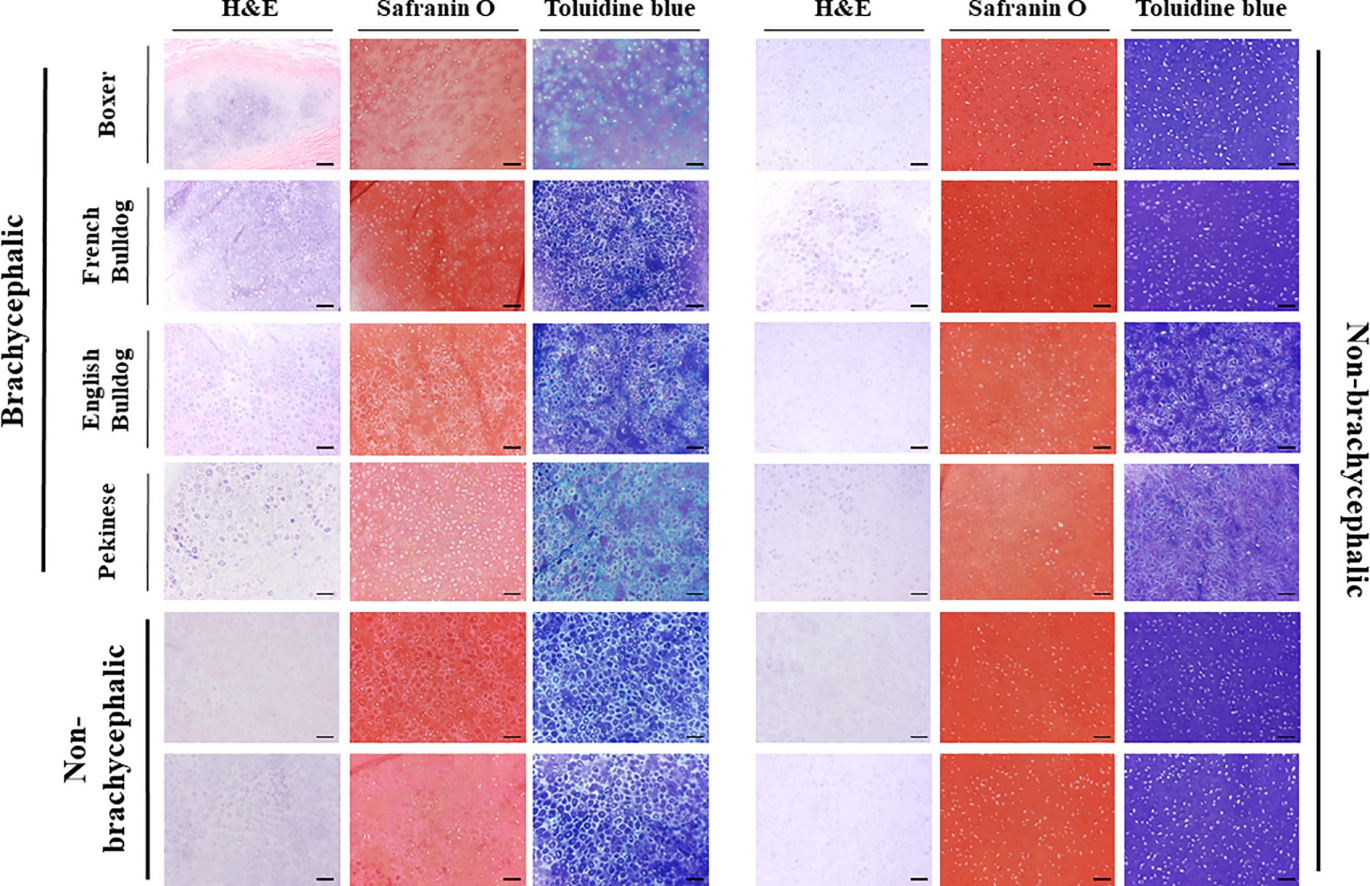
Photomicrographs of arytenoid cartilages obtained from brachycephalic and non-brachycephalic dogs. Bar = 100 μm.

Microphotographs from brachycephalic and non-brachycephalic dogs are shown in Fig 2. Histological results for the BC and the NBC group are reported in Table 2. The median total numbers of chondrocytes from 5 random areas of arytenoid cartilage at magnification of 400x in the BC group was 43.2 (range: 41–49.2) and in the NBC group was 48.7 (range: 45–55.2) (P = 0.0136). The number of degenerative chondrocytes from 5 random areas at magnification of 400x was not significantly different between the BC (median: 3.4, range: 2.6–4.2) and the NBC group (median: 1.1, range: 0.2–12) (P = 0.317). The grading of safranin O staining in the BC group (median: 2. range: 1–2) was significantly higher than that in the NBC group (median: 0.5, range 0–2) (P = 0.0329). The degree of metachromatic changes with toluidine blue staining revealed significantly higher score for the BC group (median: 2, range: 2–3) than for the NBC group (median: 0.5, range: 0–22) (P = 0.0481).

**Table 2:** Histological assessment of the arytenoid cartilages.

	Breed	Number of chondrocytes	Number of degenerative chondrocytes	Grade of safranin O	Grade of metachromasia (toluidine blue)
BC	Boxer	49.2	2.6	2	2
	Pekinese	44.4	3	2	3
	French Bulldog	41	4.2	2	2
	English Bulldog	42	3.8	1	2
Median (range)		43.2 (42–49.2)	3.4 (2.6–4.2)	2 (1–2)	2 (2–3)
NBC	Golden Retriever	46.8	7.4	1	2
	German Shepherd	47.4	12	2	2
	Walker hound	55.2	0.4	0	0
	Walker hound	52.4	0.8	0	0
	Walker hound	50	0.6	1	1
	Walker hound	45	1.4	1	1
	Walker hound	46	0.2	0	0
	Walker hound	54	2.2	0	0
Median (range)		48.7 (45–55.2)	1.1 (0.2–12)	0.5 (0–2)	0.5 (0–2)

Number of chondrocytes or degenerative chondrocytes per high power field, or score of the safranin O staining or Toluidine blue staining.

**Fig 3:**
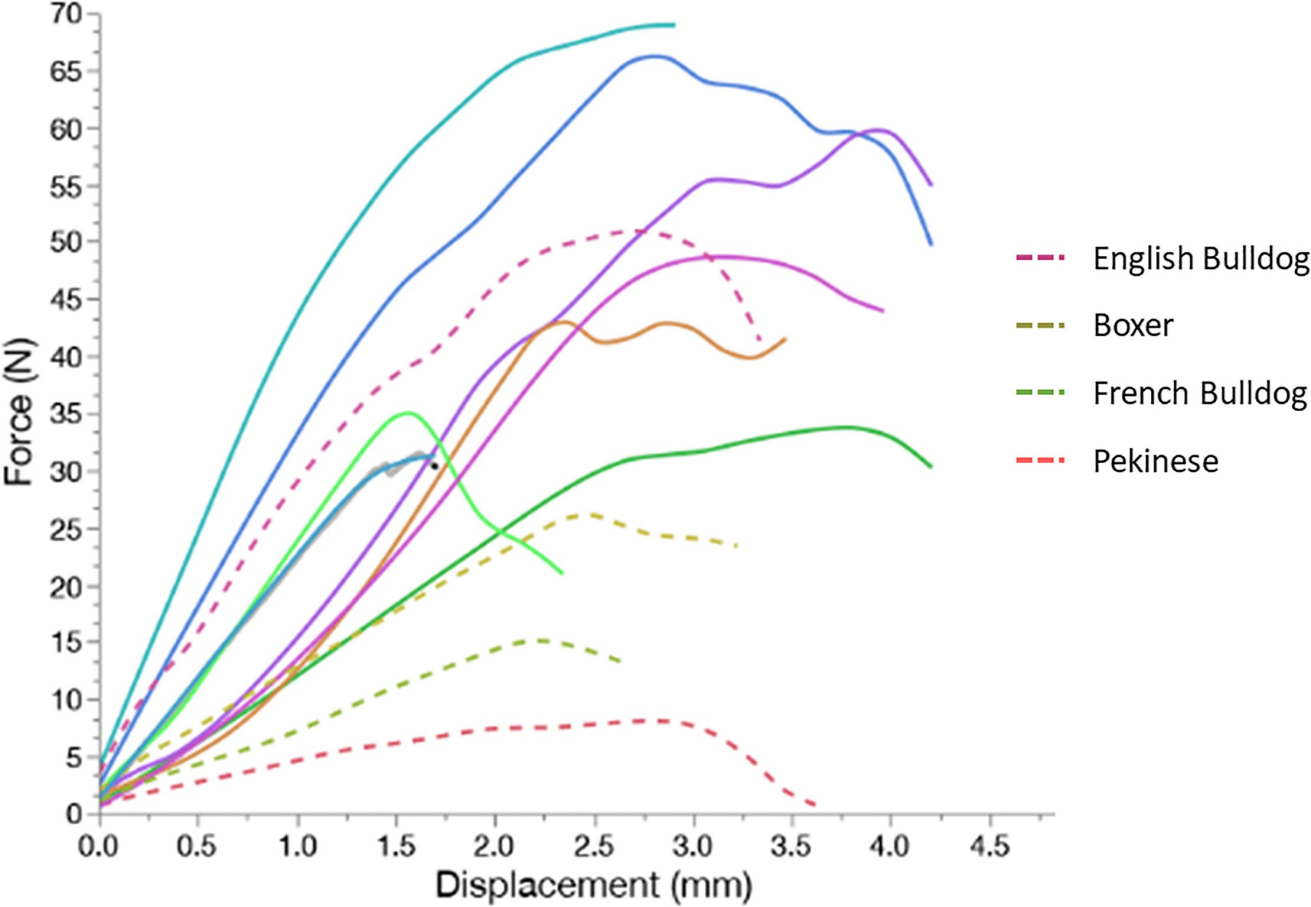
Load deformation curve for the arytenoid cartilages in the NBC group (solid line) and the BC group (dotted line).

The median load to failure of arytenoid cartilage was 27.50 N (range: 8.20–51.40 N) for the BC group and 47.20 N (range: 31.50–69.00 N) for the NBC group (P = 0.0617, power = 0.521) (Fig 3). The stiffness of the arytenoid cartilage was 6.50 N/mm (range: 3.30–18.40 N/mm) for the BC group and 19.80 N/mm (range: 11.50–23.30 N/mm; P = 0.0174) for the NBC group (Fig 3).

## Discussion

Within our study population, arytenoid cartilages of brachycephalic dogs presented degenerative histological characteristics. This was mostly demonstrated by the reduction of number of chondrocytes and the increased metachromatic changes in the arytenoid cartilages of the brachycephalic dogs. The degenerative histological characteristics translated into reduce stiffness in our sample population.

A variety of canine breeds belong brachycephalic breeds, such as Pugs, English and French bulldogs, Boston terriers, Pekingese, Maltese, Shih Tzu, Boxers, Cavalier King Charles spaniels, Yorkshire terriers, Miniature Pinscher, and Chihuahuas [4, 18, 19]. However BAS is commonly seen especially in the first three breeds and not as often in Boxer or Pekinese compared to these three breeds. Two of four dogs in the BC group of this study were Boxer and Pekinese that are brachycephalic breeds although do not commonly present with upper airway obstruction in the clinical situation [18]. Also the morphologic characteristics of the arytenoid cartilage vary greatly in different breeds [20]. This breed difference in our BC group might impact our results however the arytenoid cartilage of those dogs showed degenerative histological changes. The arytenoid cartilage of the Boxer and the Pekinese showed degenerative change via both safranin O and toluidine blue stains and a low stiffness during biomechanical testing. In addition to the difference in breeds, the age of the BC group in our study was older than most of the population reported previously. The median age in the BC group in our study was 11.5 years old while dogs in most of the studies related to brachycephalic airway syndrome are 2.6–4.5 years old [21, 22]. None of the dogs in our brachycephalic groups were euthanized because of upper airway disease while in the literature about brachycephalic airway syndrome dogs are presented at a younger age when they have clinical signs of upper airway obstruction. Our control population was from non-brachycephalic dogs without any history of airway disease. Six of those dogs were Walker hounds at 12 months of age. Those dogs were euthanized for reason unrelated to this research project. Therefore the age difference between the two groups might have influenced our results that younger age in NBC showed less degenerative change and higher mechanical strength and older age in BC showed more degenerative change and lower mechanical strength.

Degenerative change of the arytenoid cartilage in the brachycephalic dogs was demonstrated by a statistically significantly increased grade of staining with both safranin O and toluidine blue in the BC group compared to the NBC group. This grading system for arytenoid cartilage staining characteristics is subjective but has been validated to analyze cartilage repair tissue [17]. Both cationic dyes allow visualization of proteoglycans as well as glycosaminoglycans in a tissue due to their high affinity for the sulfate groups in proteoglycans of cartilage tissue. The basic proteoglycan unit consists of a core protein with one or more covalently attached glycosaminoglycan(s). Proteoglycan gives cartilage resistance to compressive loading and gives the tissue its resistance to tension [23]. Safranin O staining is proportional to proteoglycan content in normal cartilage [24]. Toluidine blue also shows intense staining due to its high affinity for the sulfur in cartilage [24] and displays a purple metachromatic color with attached to glycosaminoglycans in cartilage matrix. The degree of metachromatic color in cartilage tissue depends upon the amount of glycosaminoglycans in the cartilage tissue. Both safranin O and toluidine blue staining in this study revealed significantly higher grade in the BC group compared to the NBC group, which indicates the cartilage in the BC group contains less proteoglycans and glycosaminoglycans and more degenerative condition compared to that in the NBC group. These degenerative changes of brachycephalic arytenoid cartilage tissues in our study could have been the cause of reduced stiffness leading to mechanical weakness. However, additional approaches for assessing cartilage matrix composition and integrity including immunohistochemical staining for specific proteins, protein fragments, cleavage sites and other epitope would be required for better assessment of degeneration in arytenoid cartilage of brachycephalic dogs.

Stiffness of the arytenoid cartilage was affected in the brachycephalic dogs in this study. The load to failure was lower also in the BC group than the NBC group. Loss of stiffness and medial deviation of arytenoid cartilage have been hypothesized as a cause for obstruction of the rima glottidis [6, 7, 9, 11]. Our study supports this hypothesis since the load to failure and stiffness were lower in the BC group compared to the NBC group. Our mechanical test to assess load to failure and stiffness in this study did not reproduce the physiologic movement of arytenoid cartilage during inspiration and expiration. Therefore the anisotropic properties of the arytenoid cartilage could have affect our data. However, all the samples were tested in a similar fashion therefore it should minimize the effect of anisotropic properties of the cartilage.

It was not possible to establish if the histological and biomechanical differences established in this study could be the cause or the consequences of laryngeal collapse in brachycephalic dogs. Degenerative changes in the arytenoid cartilages could have induced weakness in the arytenoid leading to collapse, or chronic laryngeal collapse from the negative airway pressure induced a breakdown of the cartilage resulting in the degenerative changes in the arytenoid cartilages. A larger study with brachycephalic dogs of different ages with different degree of upper airway obstruction will be needed to establish a relationship between the histological and the biomechanical changes observed in this pilot study. The reduced stiffness of the arytenoid cartilages of the brachycephalic dogs might explain why the efficacy of arytenoid cartilage lateralization is questionable in dogs in suffering from chondromalacia when arytenoid cartilages have a tendency to inwardly rotate during inspiration [9, 11].

There are several limitations in this study. First of all, the small sample size of arytenoid cartilages with inconsistency of age are important factors. However even with a small population statistically significant differences were demonstrated. The morphologic characteristics of the arytenoid cartilage may vary greatly in different breeds. The load to failure showed a trend toward significance in this study. The power for that test was low suggesting a significant difference could be present with a higher number of cases. The clinical signs related to BAS or laryngeal collapse in each dog in this study were unknown. Therefore we could not correlate the severity of brachycephalic airway syndrome with any of the data collected in this study. None of the brachycephalic dogs were euthanized for reasons related to upper airway obstruction. Age could also influence our histological data since degeneration of the arytenoid cartilage in brachycephalic dogs progresses with age. Previous studies assessed soft palate histologically and demonstrated diverse muscular modifications in adult brachycephalic dogs that were not present in brachycephalic neonatal dogs supporting the hypothesis that depression during inspiratory phase causes chronic vibration and microtrauma which leads to progressive soft palate alternations with age [25, 26]. A larger study using specimens from dogs with consistent age and breed would allow better assessment of degeneration in arytenoid cartilage of brachycephalic dogs. These limitations potentially could impact both histological and mechanical evaluation.

This study suggests that arytenoid cartilage in brachycephalic dogs tend to be more degenerate than in non-brachycephalic dogs. This degenerate state could contribute to chondromalacia and mechanical weakness of arytenoid cartilage and result in source of failure after arytenoid lateralization. Our data and future study may lead to the modification of current therapy and the development of novel therapies and in brachycephalic dogs with laryngeal collapse.
